# Molecular beacon probes–base multiplex NASBA Real-time for detection of HIV-1 and HCV

**Published:** 2012-06

**Authors:** S Mohammadi-Yeganeh, M Paryan, S Mirab Samiee, V Kia, H Rezvan

**Affiliations:** 1 Biotechnology Research Center, Pasteur Institute of Iran, Tehran, Iran; 2 Molecular Biology and Genetic Engineering Department, Stem Cell Technology Research Center, Tehran, Iran; 3 Day General Hospital Laboratory, Tehran, Iran; 4 Food and Drug Laboratory Research Center, Ministry of Health and Medical Education, Tehran, Iran; 5 Department of Medical Biotechnology, Tarbiat Modares University, Tehran, Iran

**Keywords:** HIV-1, HCV, Multiplex NASBA assay, Molecular beacon

## Abstract

**Background and Objectives:**

Developed in 1991, nucleic acid sequence-based amplification (NASBA) has been introduced as a rapid molecular diagnostic technique, where it has been shown to give quicker results than PCR, and it can also be more sensitive. This paper describes the development of a molecular beacon-based multiplex NASBA assay for simultaneous detection of HIV-1 and HCV in plasma samples.

**Materials and Methods:**

A well-conserved region in the HIV-1 *pol* gene and *5’-NCR* of HCV genome were used for primers and molecular beacon design. The performance features of HCV/HIV-1 multiplex NASBA assay including analytical sensitivity and specificity, clinical sensitivity and clinical specificity were evaluated.

**Results:**

The analysis of scalar concentrations of the samples indicated that the limit of quantification of the assay was <1000 copies/ml for HIV-1 and <500 copies/ml for HCV with 95% confidence interval. Multiplex NASBA assay showed a 98% sensitivity and 100% specificity. The analytical specificity study with BLAST software demonstrated that the primers do not attach to any other sequences except for that of HIV-1 or HCV. The primers and molecular beacon probes detected all HCV genotypes and all major variants of HIV-1.

**Conclusion:**

This method may represent a relatively inexpensive isothermal method for detection of HIV-1/HCV co-infection in monitoring of patients.

## INTRODUCTION

Human immunodeficiency virus-1 (HIV-1) and hepatitis C virus (HCV) can transmit through common routes such as mother to child in pregnant women, injection drug abuse, and blood or blood products (1–4). Approaches to reduce transmission of these blood-borne agents from donor to recipient are stringent donor selection and performing screening tests to detect anti-HIV and anti-HCV seroreactivity in donors (5, 6). However, during a window period of these viruses when most serological tests are negative, infection cannot be detected in donors with newly acquired infection. Therefore, although sensitive serological tests have been developed effectively and successfully, there is still a residual risk of viral infection, due to the possibility of the HIV or HCV transmission either during the so-called window period, when viremia precedes seroreactivity, or in the presence of different viral serotypes, where the serological tests may be ineffective (5–7).

In order to reduce this residual risk, application of molecular techniques is recommended and is in use in several countries. However, molecular diagnostic technology has a major disadvantage namely high expense (1, 7). In this respect multiplex assays are developed to reduce the cost as well as running time (1, 7–10). A number of platforms of isothermal nucleic acid amplification have been developed and introduced in the past 18 years (11, 12).

**Fig. 1:**
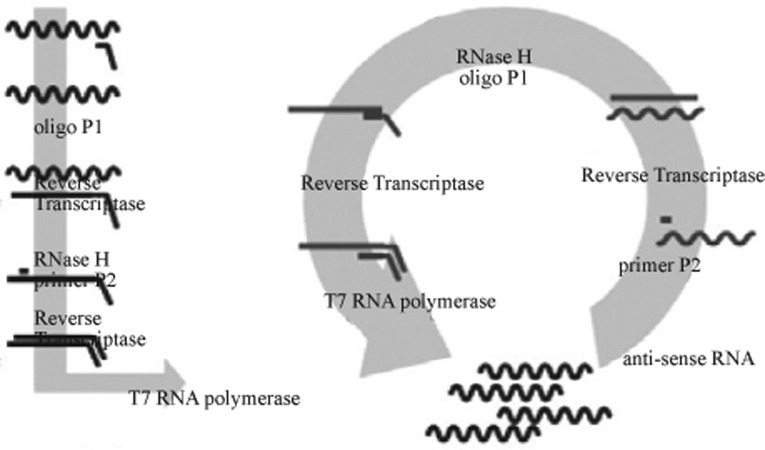
Process of the continuous, homogeneous, isothermal amplification of RNA

NASBA was selected since it is a simple and rapid alternative for nucleic acid amplification in case of RNA viruses. In this technique RNA is amplified to a billion fold in around two hours (Fig. 1) (13). It is an isothermal process and therefore no advanced equipment such as thermal cycler is required(14). The NASBA process requires fewer cycle than polymerase chain reaction (PCR) to yield a desired amplification. When using PCR, the number of molecules doubles each step, therefore it requires about 20 cycles to amplify the template to a million-fold(14). With NASBA, however, 10-100 copies of RNA are generated in each transcription step, thus only four to five cycles are required to achieve a similar amplification. As a result, both the total incubation time and the overall error frequency are lowered with NASBA. Errors that are intrinsic in some enzymatic activities (e.g. reverse transcriptase) are cumulative, and therefore fewer cycles should reduce the probability of these errors occurring (14).The objective of the present study was to develop a sensitive and rapid multiplex real-time nucleic acid sequence-based amplification (NASBA) assay to simultaneously detect HIV-1 and HCV genomes, using molecular beacon probes. This multiplex real-time NASBA was assessed using HIV-1 and HCV specific oligonucleotides that encompass RNA sequences in viral conserved regions of genome in order to yield primers able to detect all the HCV genotypes and HIV-1 major subtypes.

## MATERIALS AND METHODS

### Clinical samples

Fifty positive plasma samples, including 30 positive samples from co-infected HIV-1 and HCV patients and 20 HIV-1 and HCV negative plasma samples spiked with dilutions of transcribed RNA, were tested to verify the efficacy of the NASBA real-time assay. Twenty healthy plasma samples approved negative for HCV and HIV-1 RNA by immunoassay method, were also used to assess the specificity.

### Primers and probes design

**Fig. 2:**
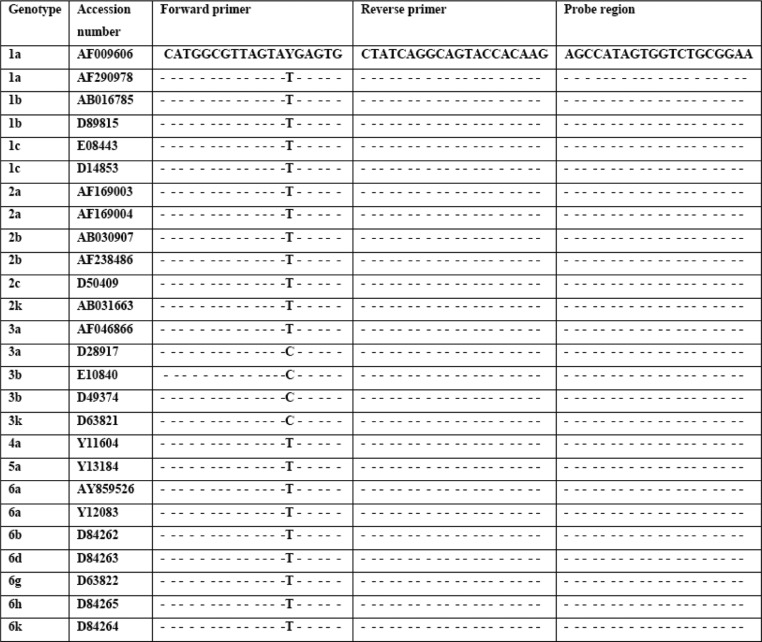
Comparison between HCV sequences of several genotypes and oligonucleotide sequences. The oligonucleotide sequences were analyzed by Mega4 software for their compatibility.

**Fig. 3:**
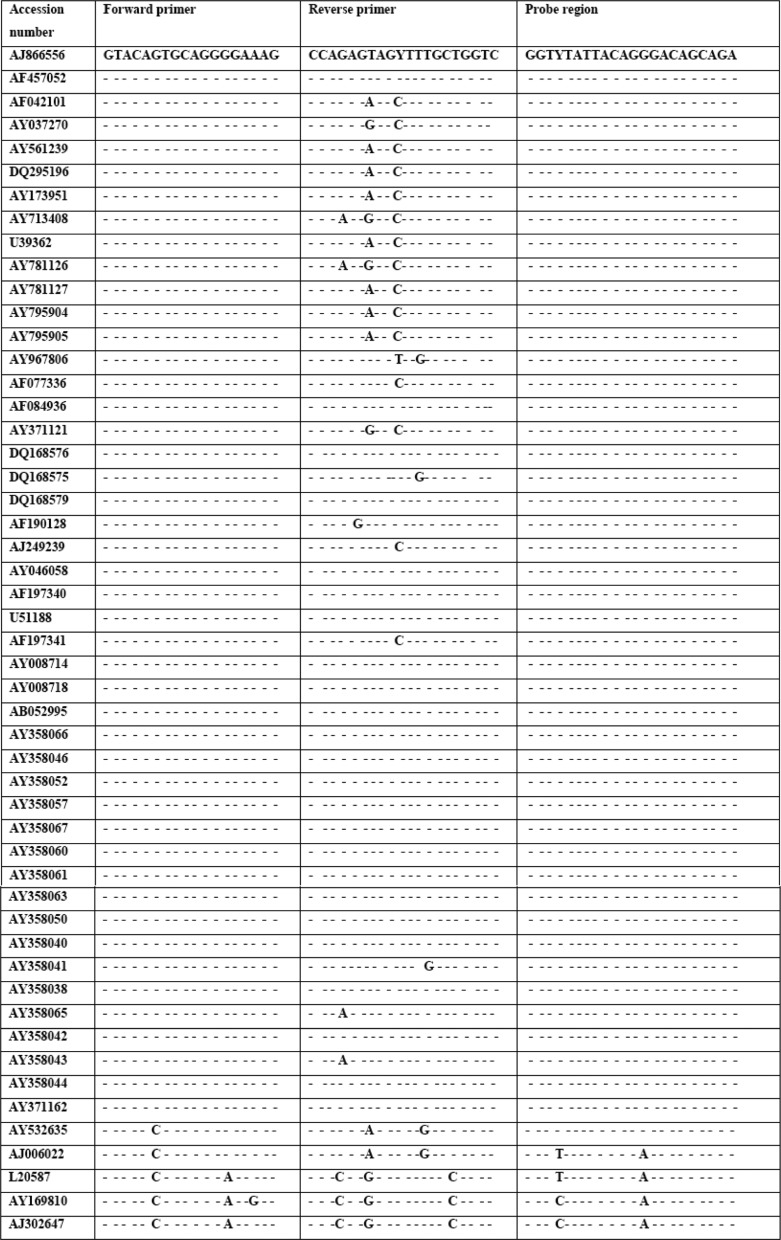
Comparison between HIV-1 sequences of several subtypes. The oligonucleotide sequences were analyzed by Mega4 software for their compatibility.

We designed two sets of primers and molecular beacon probes on *pol* gene and *5'NCR* region of HIV-1 and HCV viruses, respectively. All the conserved sequences were selected by alignment of maximum subtype and genotype sequences retrieved from the National Center for Biotechnology Information (NCBI, Bethesda, MD) GenBank database. Sequence alignments were performed using Mega4 software (Fig. 2 and 3). To ensure the multiplexed primers would have no mis-combinations we used Beacon Designer 7.0 (Premier Biosoft, Palo Alto, CA) software to confirm the sequencing reaction. The secondary structure of primers and target sequences were assessed by Mfold (http://mfold.rna.albany.edu/?q=mfold/RNA-Folding-Form). The chosen primers amplify a 179-base pair fragment of *pol* gene and 241-base pair fragment of 5'Non coding region (*5'NCR*). For distinction between the fluorescent signal of HIV-1 and HCV, sequence-specific probes were used with two different reporter dyes (Table 1).

**Table 1:** Sequences primers and probes.

Name	Sequence 5’-3’
**HIV-1 forward primer**	GTACAGTGCAGGGGAAAG
**HIV-1 Reverse primer^a^**	*AATTCTAATACGACTCACTATAGGG* CCAGAGTAGYTTTGCTGGTC
**HIV-1 Probe^b^**	TET CCCGTGGTYTATTACAGGGACAGCAGAACGGG BHQ-1
**HCV forward primer**	CATGGCGTTAGTAYGAGTG
**HCV Reverse primer**	*AATTCTAATACGACTCACTATAGGG* CTATCAGGCAGTACCACAAG
**HCV Probe**	FAM CCGATCAGCCATAGTGGTCTGCGGAAGATCGG BHQ-1

a T7 promoter is indicated with Italic

b Stem sequences are indicated with underlines

### Viral RNA extraction from plasma

Viral RNA extraction were carried out using QiaAmp RNA mini kit (Qiagen) according to the manufacturer's manual and eluted with 50µl of nuclease-free water and stored at －80 °C until use.

### Cloning of HIV-1 and HCV fragments

The amplified fragments were gel purified (Qiagen, Germany) and cloned in PTZ57 R/A cloning vector as the manufacturer's instructions (Fermentas, Germany).

### In-vitro transcription for standard preparation

**Fig. 4:**
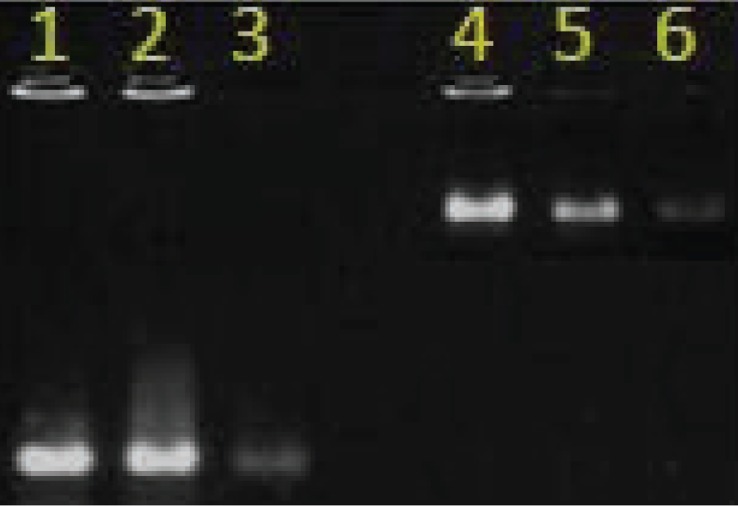
Quality of RNA transcript. Lanes 1, 2 and 3 shows RNA transcript before DNase treatment. Lanes 4, 5 and 6 shows RNA transcript after DNase treatment.

The recombinant plasmid was linearised with *EcoRI* enzyme (Fermentas, Germany). In-vitro transcription was performed in a final volume of 50 µl using the recombinant plasmid and T7 RNA Polymerase (Fermentas, Germany), in presence of 2 mM each of the ribonucleoside 5'triphosphates, 10 µl transcription buffer, 50 U RNase inhibitor and 30 U T7 RNA Polymerase in a 50 µl reaction and tube, which was incubated for 120 min at 37°C. After transcription, 5U (2U per 1 µg of DNA used) of RNase free DNase (Fermentas, Germany) was added and the tubes incubated for 30 min at 37°C to degrade the template DNA. The transcribed RNA was purified by Trizol extraction method. The integrity of RNA was checked on a 2% formaldehyde agarose gel by electrophoresis (Fig. 4) and quantitated by measuring the optical density (OD) at 260 nm.

### Multiplex NASBA Real-time

The multiplex procedure was carried out with final reaction mixture volume of 25 µl containing 40 mM Tris-HCl (pH 8.5), 50 mM KCl, 12 mM MgCl_2_, 1 mM each of the dNTPs, 2 mM each of the ribonucleoside 5'triphosphates, 10 mM dithiothreitol, and 15% (vol/vol) dimethyl sulfoxide. 0.4 µM of each primer, 0.2 µM each probe was used for the multiplex NASBA. 5 µl of purified RNA extracted from each sample was added to 18 µl of multiplex amplification mixture in a 0.2-ml microcentrifuge tube, which was incubated for 5 min at 65°C in order to disrupt secondary structures in the target RNA. Each tube was immediately cooled to 41°C for 5 min, after which 2 µl of an enzyme mixture containing 2.6 µg of bovine serum albumin (in 50% glycerol; Roche Diagnostics Corp., Indianapolis, Ind.), 40U of T7 RNA polymerase, 8 U of avian myeloblastosis virus reverse transcriptase, 0.2 U of RNase H and 12.5 U of RNasin (All enzymes were purchased from Fermentas, except AMV-reverse transcriptase from Roche) were added.

Microtubes were transferred to a Rotor-Gene 3000 (Corbett Research, Australia). Development of fluorescence was followed in closed tubes for 90 min at 41°C. Fluorescence intensity data were recorded every minute of the NASBA reaction. Moreover, NASBA amplification products were analyzed at the end of the amplification step, by electrophoresis of 5 µl of reaction on a 2% agarose gel containing 0.5 mg/ml EtBr. Gels were run at 100 V for 30 min in TAE buffer (40 mM Tris-acetate, 1 mM EDTA, pH 8.0). Bands were visualized by UV excitation and photographed.

### Sensitivity and specificity

To determine the analytical specificity, we performed BLAST using NCBI BLAST program. To determine the clinical specificity, some viruses(e.g. HBV, HTLV-1, B19, HSV-1, HSV-2, HHV-6, HHV8, HCMV and EBV) and 20 negative samples were tested. These samples were previously tested for HIV-1 and HCV using immunoassay method. To determine the analytical sensitivity, the transcribed RNA mentioned above was used. A serial dilution of each standard from 10^4^-50 copies/ml was prepared. We used the instruction in URI Genomics & Sequencing Center (http://www.uri.edu/research/gsc/resources/cndna.html) to convert optical density of transcribed RNA to copy number. To estimate the minimal detection limit, 10-fold serial dilutions of viral RNA samples were made in DNase and RNase free water. To assess the clinical sensitivity, fifty positive samples were tested to verify the efficacy of the NASBA real-time assay.

## RESULTS

### Multiplex real-time NASBA for detection of HIV-1 and HCV

**Fig. 5:**
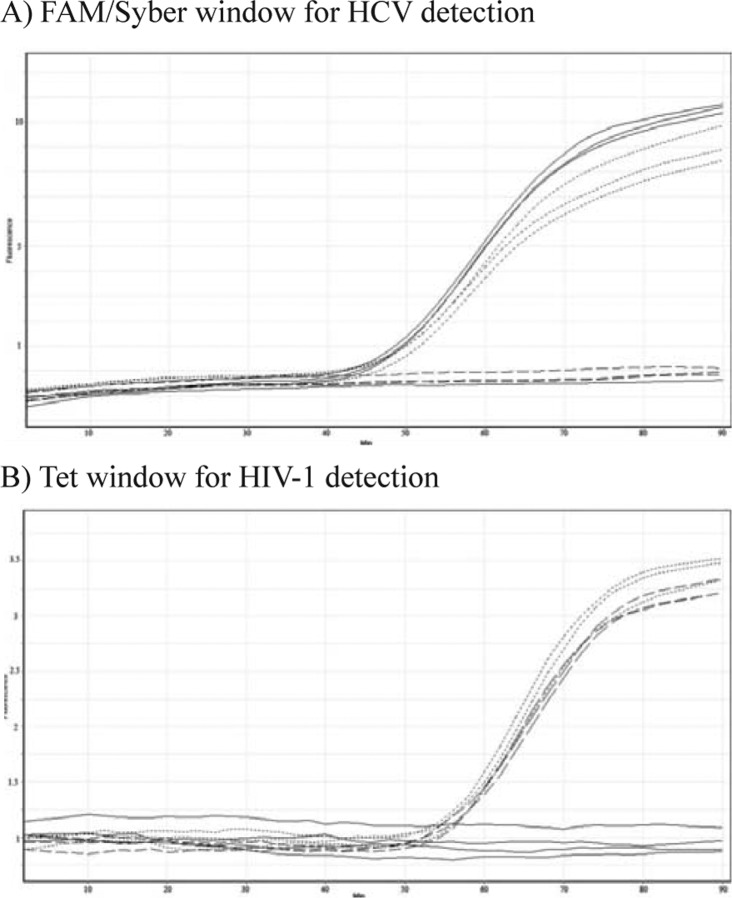
Real-time NASBA detection of two different viral genomes in a multiplex format. Each reaction contained two sets of NASBA primers specific for unique HIV-1and HCV nucleotide sequences and two molecular beacons, each specific for one of the two amplicons and labeled with a differently colored fluorophore. HCV is plotted in Solid, Multiplex format plotted in Dotted, HIV is plotted in Dashed and Non template control is plotted in thin line.

Two molecular beacons with different fluorophore labels were designed to distinguish between HIV-1 and HCV viruses (Fig. 5a and b). These probes recognized the *5'NCR* region of HCV and *pol* gene of HIV-1, they showed successful specific hybridization with their respective target sequences during NASBA amplification. For each virus, amplification led to a rise in fluorescence that was characteristic of the labeled fluorophores attached to their respective *5'NCR* region or *pol* gene sequence. The sequence of the beacons and the amplification primers were selected due to showing better sequence conservation in comparison with other regions or genes. Nevertheless, the few mutations observed in some genotypes of HCV and subtypes of HIV-1 were not located at critical positions of the primers and probe, such as the 3′ and 5′ end of the primers and probe, respectively.

**Fig. 6:**
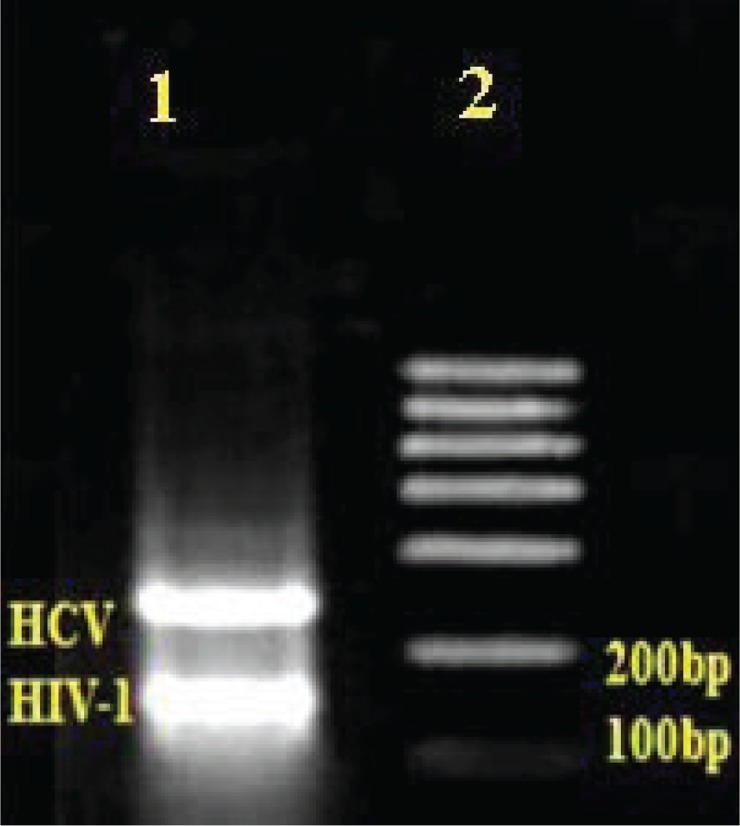
Shows multiplex NASBA amplification. 241 bp and 179 bp bands in lane1 show simultaneous amplification of HCV and HIV-1, respectively. Lane 2 is a 100 bp RNA marker (Fermentas#SM1831, RNA Ladder 100-1000bp).

As shown in (Fig. 6), agarose gel electrophoresis confirmed the exact length of the amplicons: two bands of 179bp and 241bp, specific for HIV-1 and HCV, respectively, were detected in all of the co-infected samples analyzed.

### Analytical sensitivity and specificity assay

For HIV-1/HCV viruses dilutions from transcribed RNA were tested in multiplex real-time NASBA assay from 10^4^ to 50 copies/ ml. To estimate the analytical sensitivity, serial dilutions of each virus were prepared. The analytical sensitivity of assay was 1000 copies/ml for HIV-1 and 500copies/ ml for HCV. Moreover, the limit of quantification assay was <1000 copies/ml for HIV-1 and <500 copies/ ml for HCV with 95% confidence interval (Table 2).

**Table 2:** Limit of quantification Multiplex Real-time NASBA assay for HIV-1^a^/HCV^b^ detection

aHIV-1 concentration (copies/ml)	Number tested	Number positive	Positive rate (%)
10^4^	10	10	100
10^3^	10	10	100
500	10	3	30
50	10	0	0

bHCV concentration (copies/ml)	Number tested	Number positive	Positive rate (%)
10^4^	10	10	100
10^3^	10	10	100
500	10	10	100
50	10	7	70

a The LoQ for HIV-1 was determined to be 1000 copies/ml with 95% confidence interval.

b The LoQ for HCV was determined to be 500 copies/ml with 95% confidence interval.

Cross-reactivities were not observed when HBV, HTLV-1, B19, HSV-1, HSV-2, HHV-6, HHV8, HCMV and EBV samples were tested by the multiplex Real-time NASBA assay (data not shown) that confirmed the specificity.

### Clinical sensitivity and specificity

49 samples out of 50 positive samples were positive for both viruses and one sample was positive only for HCV. Multiplex real-time NASBA assay showed a 98% sensitivity and 100% specificity.

## DISCUSSION

The exigency for molecular diagnostic tests for blood donations in addition to some of the currently licensed serological methods is obvious because of the ability of these assays to remarkably reduce the window period during which serological methods fail to detect the infection (15–17). A negative result by serological methods may be a false-negative result if the donor has been recently infected.

Conventional diagnostic methods such as immunological and serological tests have lower sensitivity than molecular techniques such as the PCR (18, 19). Nevertheless, the cost and necessity for technical expertise makes application of PCR more difficult for laboratories in developing countries and rural areas(18).

NASBA is a homogeneous and direct RNA amplification process which has been shown previously to have a sensitivity comparable to PCR(18). No thermocycler is required and a standardized constant reaction temperature is applied for highly reliable amplification of a RNA target. Due to reduction in expenses, the multiplex HIV-1/HCV NASBA assay could also be applied as a confirmatory technique for detection of these viruses.

Previous studies suggested molecular tests as a replacement for commercially available seroconversion panels (20, 21). Although there is not any report available for HIV-1 /HCV multiplex NASBA assay, this technique wildly used for detection of each virus.

The commercial kits for HIV-1 detection are accessible. The NucliSens^®^ HIV-1 QT is an in vitro nucleic acid sequence based amplification test for the quantitation of HIV-1 RNA in human plasma. The test can quantitate HIV-1 RNA over the range of 176 to 3.47X10^6^ copies/mL. The test is intended for use in conjunction with clinical presentation and other laboratory markers of disease progression for prognostic assessment of HIV-1 infected patients, and for monitoring the effects of anti-retroviral therapy by serial measurements of plasma HIV-1 RNA for pediatric and adult patients with baseline viral loads greater than 93,000 and 28,000 copies of HIV-1 viral RNA/ml, respectively(www.biomerieux-diagnostics.com).

Workenesh Ayele, et al. [2004] developed a Nucleic Acid Sequence-Based Amplification assay that used gag-based molecular beacons to distinguish between HIV-1 subtype C and C’ infections in Ethiopia. This assay is country specific for HIV-1 subtype C epidemics and it will contribute to characterizing the circulating viruses in this population, thereby generating the information necessary for the development of a potential efficacious HIV-1 appropriate vaccine(22).

In another experiment Rute Antunes, et al. evaluated the clinical sensitivitiy of three viral load assays with plasma samples from a pediatric population predominantly infected with HIV-1 subtype G and BG recombinant forms. To determine the viral load, AMPLICOR HIV-1 Monitor Test 1.5, Nuclisens HIV-1 QT, and Quantiplex HIV RNA 3.0 (bDNA) were investigate. AMPLICOR v1.5 and Quantiplex v3.0 detected all positive samples with a good correlation. Thirty-eight out of 61 specimens containing HIV-1 subtype B, G, or recombinant BG, could not detected by Nuclisens HIV-1 QT (23).

We have described a multiplex assay in which molecular beacons are used to detect amplicons in real-time NASBA under homogeneous conditions. In this assay, the hairpin stem of the molecular beacons keeps the quencher and the fluorophore at such close proximity to each other that quenching occurs as a result of physical contact between the two labeled moieties.

The multiplex NASBA assay for detection of HIV-1/HCV has advantages with respect to time and cost in comparison to assays designed with a single-target assay. Using multiplex aligning sequences in nucleotide database of NCBI (www.ncbi.nlm.nih.gov/nucleotide), two sets of primers and molecular beacon probes on the most conserved region of *pol* gene of HIV-1 and *5'NCR* region of HCV were designed.

The data presented in this paper demonstrated that these primers and probes match the well-conserved sequences among different HCV genotypes as well as major HIV-1 subtypes. In fact, genotype independent detection of any virus such as HIV-1 and HCV is reliant upon locating the most conserved region of viruses genome by using a complete databank of aligned sequences for genome of both viruses.

This study demonstrates that the multiplex real-time NASBA assay using the appropriately designed molecular beacon probes has 98% clinical sensitivity and 100% specificity for the detection of HIV-1/HCV from plasma samples. Based on the analytical sensitivity data, the multiplex real-time NASBA assay showed significantly lower limit of quantification for HCV (<500 copies/ml) than that of HIV-1 (<1000 copies/ml) with 95% confidence interval and no cross-reactions were observed when other blood-borne viruses were tested by the multiplex real-time NASBA assay.
